# Optical Fiber-Tip Sensors Based on In-Situ µ-Printed Polymer Suspended-Microbeams

**DOI:** 10.3390/s18061825

**Published:** 2018-06-05

**Authors:** Mian Yao, Xia Ouyang, Jushuai Wu, A. Ping Zhang, Hwa-Yaw Tam, P. K. A. Wai

**Affiliations:** 1 Photonics Research Centre, Department of Electronic and Information Engineering, The Hong Kong Polytechnic University, Hong Kong, China; mian.jr.yao@connect.polyu.hk (M.Y.); alex.wai@polyu.edu.hk (P.K.A.W.); 2 Photonics Research Centre, Department of Electrical Engineering, The Hong Kong Polytechnic University, Hong Kong, China; xia.ouyang@connect.polyu.hk (X.O.); jushuai.wu@connect.polyu.hk (J.W.); eehytam@polyu.edu.hk (H.-Y.T.)

**Keywords:** optical fiber-tip sensors, optical 3D µ-printing, refractive index sensors, gas-pressure sensors, lab-on-fiber

## Abstract

Miniature optical fiber-tip sensors based on directly µ-printed polymer suspended-microbeams are presented. With an in-house optical 3D μ-printing technology, SU-8 suspended-microbeams are fabricated in situ to form Fabry–Pérot (FP) micro-interferometers on the end face of standard single-mode optical fiber. Optical reflection spectra of the fabricated FP micro-interferometers are measured and fast Fourier transform is applied to analyze the cavity of micro-interferometers. The applications of the optical fiber-tip sensors for refractive index (RI) sensing and pressure sensing, which showed 917.3 nm/RIU to RI change and 4.29 nm/MPa to pressure change, respectively, are demonstrated in the experiments. The sensors and their optical µ-printing method unveil a new strategy to integrate complicated microcomponents on optical fibers toward ‘lab-on-fiber’ devices and applications.

## 1. Introduction

Optical fiber sensors have received remarkable successes in a wide range of applications—such as inertial navigational systems, environmental and structural monitoring, biochemical sensing, healthcare, food industry, and homeland security—because of their small size, electromagnetic interference (EMI) immunity, high sensitivity, remote sensing, and multiplexing capabilities [1,2,3]. Recently, with the development of micro-/nano-technology, optical fiber-tip sensors integrated with functional materials and microscale components have attracted considerable attention [4,5,6]. It is because an optical fiber end-face is an inherently light-coupled substrate [5], which provides an ideal platform for development of compact and highly integrated photonic devices and sensors stepping toward a new horizon of ‘lab-on-fiber’.

A number of optical fiber-tip sensors with various structures and working mechanisms were proposed. For instance, one of the widely used structures in optical fiber-tip sensors is Fabry–Pérot (FP) interferometers, which are typically composed of a suspended diaphragm to form an FP cavity on optical fiber ends. Because of their simple structure and high sensitivity, FP cavity-based fiber-tip sensors have been intensively investigated for detection of various physical and biological parameters, such as pressure [7,8,9], temperature [10], acoustic wave [11], and refractive index (RI) [12]. If the reflectivity of the mirrors of such FP cavities is increased, optical microresonators can be formed on the end face of optical fiber for e.g., ultrasound sensing [13]. Moreover, localized surface plasmon resonance (LSPR) biochemical sensors were fabricated by patterning periodic gold nanodot arrays [14], and high-performance surface-enhanced Raman scattering (SERS) sensors were demonstrated by capping optical fiber end-faces with multilayer silver nanoparticles [15]. 

However, the challenge is that the tiny size and large aspect-ratio of optical fibers make the fabrication of optical fiber-tip devices difficult by using conventional microfabrication technologies. Although a diversity of fabrication techniques—such as photolithography [16], nanoimprinting [17], interference lithography [18], electron-beam lithography [19], focused ion-beam milling [20], multiphoton polymerization [21,22,23,24,25]—have been proposed to overcome this challenge, most of them have common drawbacks of being time consuming, having material specificity, and lacking flexibility.

**Figure 1:**
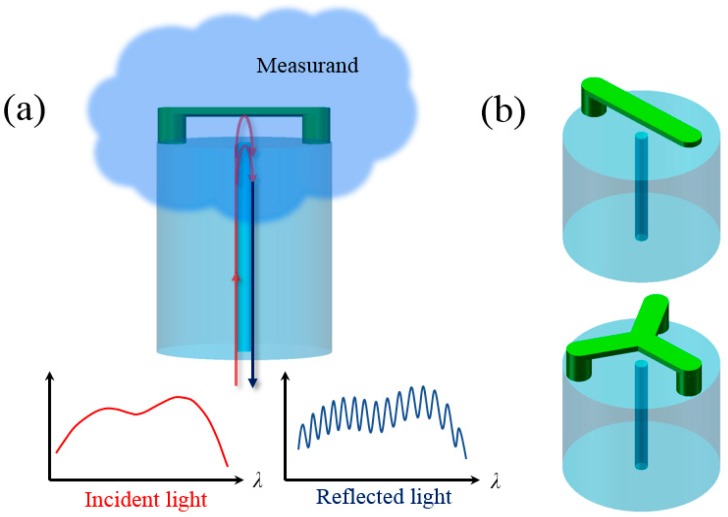
(**a**) Schematic of an optical fiber-tip sensor and its working principle. (**b**) Schematics of the other two optical fiber-tip sensors with different structures of suspended microbeams.

Recently, we demonstrated that suspended-mirror devices (SMDs) can be directly fabricated on the end face of fiber-optic ferrules by using an optical 3D μ-printing technology [26]. However, such ferrule-top SMD sensors are still too large for applications where the sensors need to be deployed into very small space such as microfluidic channels and blood vessels. In this paper, we present an improved optical µ-printing technology to directly fabricate suspended-microbeams on the end face of a standard single-mode optical fiber. Figure 1a depicts the structural design and the working principle of the optical fiber-tip sensor based on suspended-microbeams. The suspended microbeam on optical fiber end-face forms a fiber-top air cavity. As a result, optical interference occurs between the light waves reflected from the interface between fiber end-face and air, and the interfaces between air and the two surfaces of the suspended microbeam. If the device is immersed into a liquid or gas whose RI is lower than the indices of glass and the polymer, it can be used to monitor the change of the RI of the liquid or gas through monitoring the shift of the interference fringe of reflection spectrum. As shown in Figure 1b, various suspended-microbeams with different geometries can be designed to meet the needs of diverse sensor applications.

## 2. Materials and Methods

### 2.1. Materials

EPON resin SU-8 (Momentive Ltd., Waterford, NY, USA) was used in the fabrication of suspended-microbeams because of its good properties including highly transparent in both visible and near infrared band range, chemical resistance, and good mechanical strength. The refractive index of photopolymerized SU-8 at the wavelength of around 1550 nm is 1.57 [27].

Octoxyphenylphenyliodoniumhexafluoroantimonate (OPPI) (Hampford Research Inc., Stratford, ON, Canada) and tributylamine (Meryer Chemical Technology Co., Ltd., Shanghai, China) were used as photoacid generator and inhibitor, respectively. 2-(2H-Benzotriazol-2-yl)-4,6-bis(1-methyl-1-phenylethyl)phenol (i.e., Tinuvin 234) (Sigma-Aldrich Inc., St. Louis, MO, USA) was adopted as UV absorption agent to control light penetration depth so as to enhance the vertical distinguishability in the printing process. These compositions were dissolved by cyclopentanone (Sigma-Aldrich Inc., St. Louis, MO, USA) in a weight ratio of OPPI/tributylamine/Tinuvin 234/SU-8 = 2:0.014:0.2:100. Propyleneglycol monomethylether acetate (PGMEA) (Sigma-Aldrich Inc., St. Louis, MO, USA) was used as developer.

### 2.2. Optical 3D μ-Printing Processes

**Figure 2:**
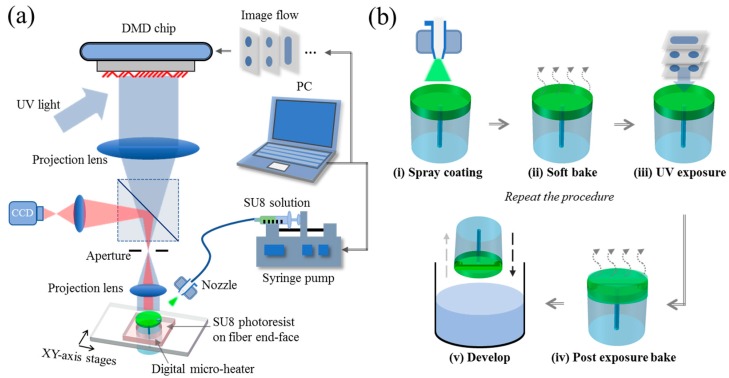
(**a**) Schematic diagram of the optical 3D µ-Printing technology. (**b**) Flow chart for printing the optical fiber-tip sensors.

An in-house optical exposure setup, as shown in Figure 2a, was used to fabricate the optical fiber-tip sensors. The setup consists of a high-power UV source (365 nm), a UV-grade digital mirror device (DMD) for generation of optical patterns, projection optics for scaling down optical images, and a digital camera for machine vision metrology [28,29,30]. As it is a vitally important step to deposit uniform thin layers of SU-8, an ultrasonic nozzle was utilized to integrate the spray coating process with the optical maskless exposure technology to establish an optical 3D μ-printing technology. An *xy*-axis motorized stage was used to precisely align the optical fiber to right positions for UV exposure and SU-8 film deposition, respectively. The thickness of the single-layer SU-8 film can be tailored by adjusting the pumping rate of the syringe pump and the scanning velocity as well as the gas pressure associated with ultrasonic nozzle and the distance between the nozzle and substrate. In order to evaporate the solution after spray coating, ceramic heaters and thermal-couple were embedded in the mount of optical fiber to form a miniature integrated digital microheater. 

To fabricate a 3D microstructure, as shown in Figure 2b, the optical fiber was firstly moved to a position below the ultrasonic nozzle for spray coating of a thin layer of SU-8. Then the film was in situ soft-baked to remove solvent. The soft-bake time was optimized according to the concentration of SU-8 solution and the film thickness. After soft baking, the sample was moved to the pre-aligned position for optical exposure, with the assistance of the digital camera-based machine vision metrology. Thereafter, the image data that was sliced from the CAD model of the 3D microstructure by self-developed add-on software was used to generate optical patterns to irradiate the SU-8 film on the optical fiber end-face. The typical exposure time was about 10 s which is associated with the power density of 35.86 mw/cm^2^. After exposure, the sample was post-baked in situ by using the integrated digital micro-heater. The processes were automatically repeated for the fabrication of the next layer of 3D microstructure. Finally, the sample was developed by using PGMEA and the developing time was about 15 min. 

## 3. Results

### 3.1. Fabrication Results

**Figure 3:**
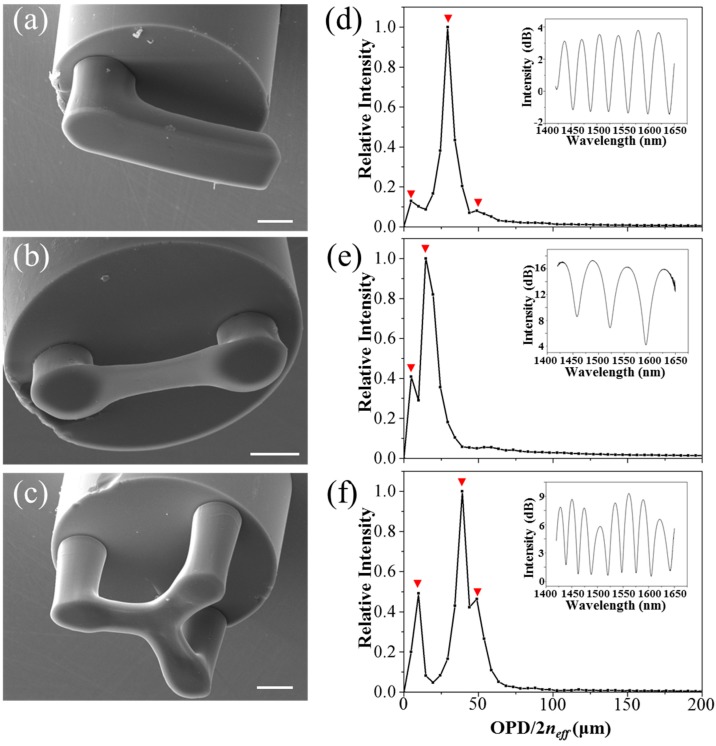
(**a**), (**b**), and (**c**) Scanning electron microscope (SEM) images of SU-8 suspended-microbeams printed on the end face of optical fibers. All scale bars are 20 μm. (**d**), (**e**), and (**f**) FFT’s results of the corresponding reflection spectra of optical fiber-tip sensors shown in (**a**), (**b**), and (**c**). Insets are the reflection spectra measured in air.

Figure 3a–c show the scanning electron microscope (SEM) images of three SU-8 suspended-microbeams fabricated on the end-faces of optical fibers. From the SEM images, the measured thicknesses of the three suspended-microbeams are 12.2, 1.0, and 6.9 μm, respectively, and the cavity lengths between the optical fiber and suspended-microbeams are 30.9, 15.6, and 40.4 μm, respectively.

### 3.2. Reflection Spectra

**Figure 4:**
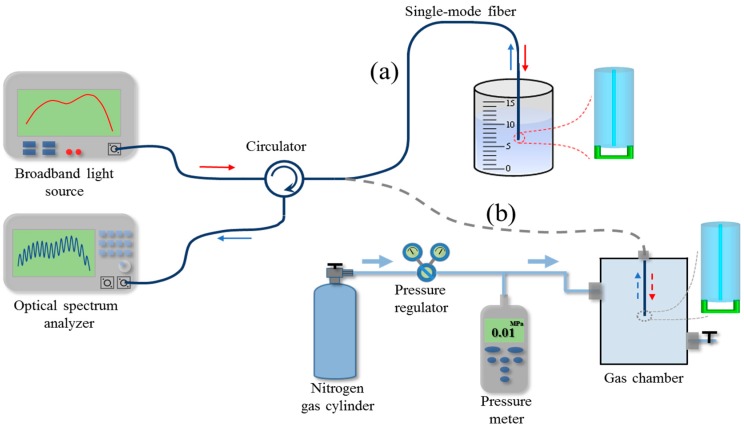
Schematic of the experimental setup for testing the optical fiber-tip sensors. (**a**) Refractive index sensing; (**b**) gas-pressure sensing.

A broadband light source, a circulator, and an optical spectrum analyzer (OSA) were used to measure the reflection spectra of the fabricated optical fiber-tip FP micro-interferometers, as shown in Figure 4. Fast Fourier transform (FFT) of the measured optical spectra was calculated to analyze the cavity information of FP micro-interferometers. Reflection spectra and their FFT results of the optical fiber-tip FP micro-interferometers are shown in Figure 3d–f, respectively. It can be seen that the positions of their highest peaks in FFT results are well accordance with the length of the air cavities shown in the SEM images. For the FP cavities with thick suspended-beams, as shown in Figure 3d,f, there are three peaks in the FFT results, which are consistent with previous SU-8 FP cavities fabricated on fiber-optic ferrules [26]. For the FP cavity with thin suspended diaphragm, as shown in Figure 3e, however, the peaks tend to merge into one peak, which avails to depress the fluctuation of inference fringe in reflection spectrum. The cavity lengths deduced from the main peaks of the FFT results are 29.3, 14.7, and 39.1 μm, respectively, which agree well with the counterparts measured by SEM images.

### 3.3. Refractive Index Sensing

One of promising applications of the optical fiber-tip sensors is to sense the refractive index of liquids. It is known that the wavelength of a resonance dip in the interference spectrum of an FP cavity can be expressed as

(1) $$\lambda_{k}=2nL/k$$

where *n* is the refractive index of the medium in cavity, *L* is the cavity length, and *k* is the order of the spectral dip. Therefore, the tracked spectral dip will shift to longer wavelength when the refractive index of measurand liquid increases. For a small change of refractive index Δ*n*, the shift of a specific spectral dip is

(2) $$\Delta \lambda ≅\left(\lambda_{k}/n\right)\Delta n$$

**Figure 5:**
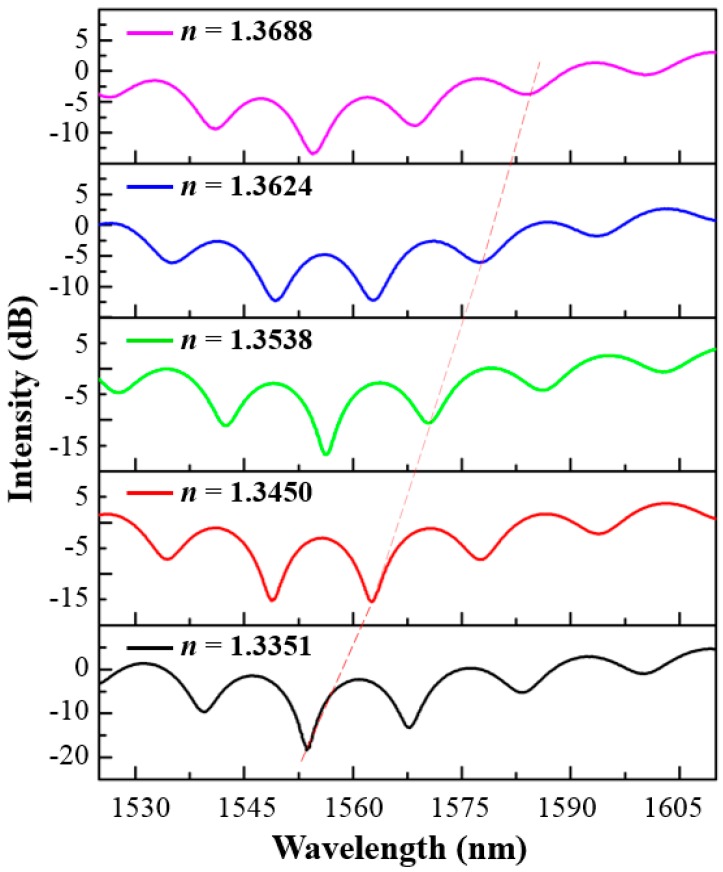
Measured reflection spectra of the optical fiber-tip sensor immersed into liquids with different refractive indices.

The response of the fabricated optical fiber-tip sensor to the change of the RI of surrounding liquids was measured by using the setup shown in Figure 4a. CaCl_2_ solutions with different concentrations were used as the testing liquids, whose refractive indices were calibrated by using a commercial refractometer. After measurement of each sample, the sensor was rinsed with deionized water and then dried in nitrogen flow. The measured reflection spectra of the sensor in different liquid samples are shown in Figure 5. The spectral dip located at 1553.7 nm when the refractive index of solution is 1.3351 was monitored, as marked by the dash line. A red shift of the spectral dip was observed with the increment of the refractive indices of the liquid samples.

**Figure 6:**
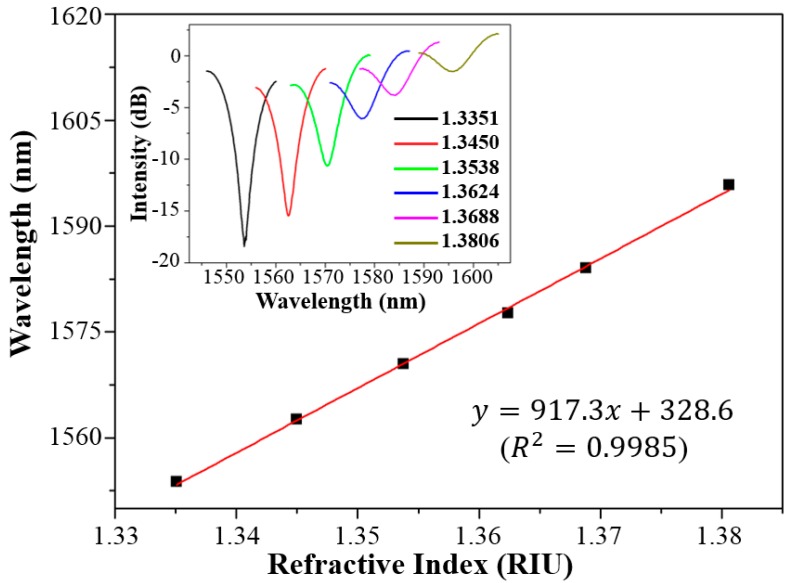
Response of a spectral dip of the optical fiber-tip sensor to the RI change of surrounding liquid. Inset shows the spectrum evolution of the spectral dip under monitoring.

Figure 6 shows the wavelength shift of the spectral dip with respect to the RI of liquids. The sensitivity of the optical fiber-tip RI sensor was calculated by linear regression to be 917.3 nm/RIU, which is close to the theoretical value of 1159.4 nm/RIU predicted by using Equation (2). Compared with other optical evanescent field-based refractive index sensors [31,32], this open-cavity optical sensor has much higher sensitivity. Nevertheless, the spectral dip becomes shallower gradually with the increase of the liquid’s RI because of the weakening of Fresnel reflections at the interfaces.

### 3.4. Gas-Pressure Sensing

The optical fiber-tip sensor can also be used to remotely monitor gas pressure in very small space. At room temperature (20 °C–25 °C), the RI of air is known as a function of pressure *p* (Pa) and temperature *t* (°C) as [33,34,35]

(3) $$n_{air}=1+2.8756\times 10^{-9}\times p\frac{1+10^{-8}(0.601-0.00972\times t)\times p}{1+0.003661\times t}$$

The quadratic term *p*^2^ can be ignored in case that the air pressure is below 1 MPa. If the cavity length is assumed as a constant, the wavelength shift of the spectral dip of *k^th^* order interference fringe with respect to the pressure change is thus

(4) $$\Delta \lambda ≅\alpha \frac{\lambda_{k}}{n_{air}}\Delta p$$

where the coefficient *α* is

(5) $$\alpha =\frac{2.8756\times 10^{-9}}{1+0.003661\times t}$$

The coefficient α is 2.679 × 10^−9^/Pa at room temperature. The above equations revealed that there is an approximately linear relationship between the wavelength shift of the optical fiber-tip sensor and air pressure at a constant temperature.

**Figure 7:**
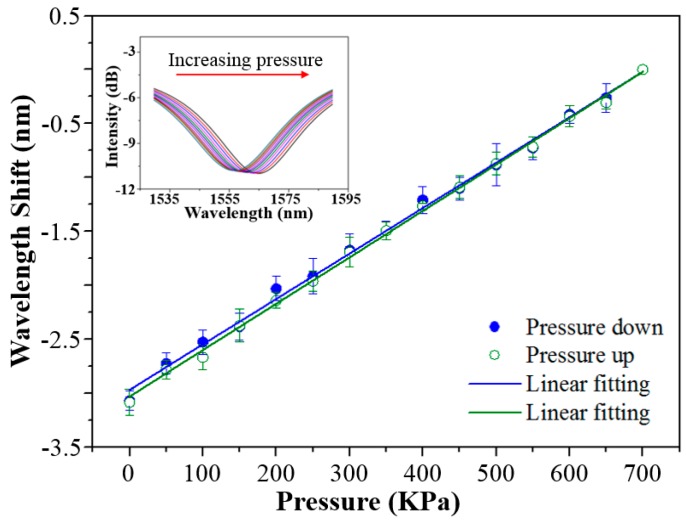
Response of a spectral dip of the optical fiber-tip sensor to the gas-pressure change of ambient environment. Inset shows the spectrum evolution of the tracked spectral dip.

The response of the optical fiber-tip sensor to the change of gas pressure was measured by using a gas chamber, whose gas pressure was controlled by a high-pressure nitrogen cylinder with gas flow regulator, as shown in Figure 4b. The gas pressure can be tuned from 0 to 700 KPa with a step of 50 KPa. A commercial pressure meter was used as to monitor the gas pressure in the chamber. Figure 7 shows the measured response of the optical fiber-tip pressure sensor to the change of gas pressure. With the increase of chamber pressure, a red shift of the spectral dip was observed because of the increase of refractive index of nitrogen gas. The optical fiber-tip sensor showed good linearity and reversibility with both increase and decrease of the gas pressure. According to the linear regression, the sensitivity of the optical fiber-tip sensor to the gas-pressure change is 4.29 nm/MPa, which is close to the theoretical value of 4.17 nm/MPa predicted by Equation (4). With the calculated noise level (i.e., 0.031 nm), the detection limit of the optical fiber-tip gas-pressure sensor is estimated to be about 22.2 KPa at a signal–noise ratio of 3 [36]. Compared with other diaphragm-based optical fiber-tip pressure sensors, this open-cavity optical sensor has relatively low sensitivity, but a wide measurement range, and thus is suitable for high air-pressure measurement applications.

## 4. Conclusions

In summary, we have demonstrated a miniature optical fiber-tip sensor by directly printing polymer suspended microbeams on the end face of standard single-mode optical fiber. The reflection spectra of the fiber-tip devices have been measured and used to analyze the Fabry–Pérot (FP) cavities formed by suspended microbeams. The optical fiber-tip sensors have been demonstrated to detect the RI change of liquid and the gas pressure of ambient environment, respectively. High sensitivities of 917.3 nm/RIU to RI change and 4.29 nm/MPa to gas-pressure change have been achieved. Such ultra-small optical fiber-tip sensors with remote sensing capability are very promising in microfluidic biosensing and environmental monitoring applications.
